# Evaluating a Digital Mental Health Tool for Implementation Into New Zealand’s Integrated Primary Mental Health and Addictions Service: Usability Study

**DOI:** 10.2196/84412

**Published:** 2026-06-04

**Authors:** Vincent Allen, Danielle Lottridge, Karolina Stasiak

**Affiliations:** 1 Department of Psychological Medicine, Faculty of Medical and Health Sciences, University of Auckland, Building 501, 85 Park Road, Grafton, Auckland, 1023, New Zealand, +64 93737599; 2 School of Computer Science, Faculty of Science, University of Auckland, Auckland, New Zealand

**Keywords:** digital mental health, usability testing, technology adoption, primary health care, integrated care scalability, integrated primary mental health and addictions model, health care workflow optimization, human-centered design, more-than-human design, context-sensitive design, user-centered design

## Abstract

**Background:**

Rising global demand for mental health services strains traditional care models, a trend evident in New Zealand, where the Integrated Primary Mental Health and Addictions (IPMHA) model was introduced to improve access to care. While the IPMHA model shows promise, significant service-delivery challenges undermine its scalability. Digital mental health tools (DMHTs) present an opportunity for digital optimization, yet their effectiveness is often limited by low practitioner adoption, a persistent implementation barrier. To ensure these tools are impactful, a user- and context-centered DMHT design approach may help mitigate practitioner adoption barriers and create solutions that can be seamlessly integrated into clinical workflows.

**Objective:**

Reporting on the Test stage of the 5-stage Design Thinking framework, this study aimed to evaluate the usability and acceptability of a DMHT software prototype intended to support health improvement practitioners working within New Zealand’s IPMHA model.

**Methods:**

Five health improvement practitioners from a single primary health organization participated in semistructured usability interviews. Data were collected using a think-aloud protocol during mock clinical sessions and analyzed using affinity diagramming to identify key software feature requirements necessary to promote usability, adoption, and workflow integration.

**Results:**

Feedback was obtained for all software minimum viable product features. While practitioners found the clinical support features valuable, 2 system-level requirements were identified as prerequisites for adoption. The first was administrative optimization: the DMHT must reduce workload by automating tasks, such as clinical note entry, data reporting, and psychometric scoring. The second was seamless integration with existing clinic patient management software to eliminate double-handling of data and resolve IT-related workflow frustrations.

**Conclusions:**

For a DMHT to be successfully adopted by IPMHA practitioners, it must primarily function to solve existing administrative and workflow inefficiencies. Clinical support features, such as the provision of therapeutic tools and exercises, though helpful, are secondary to the tool’s ability to be a practical, efficient, and fully integrated component of daily practice. These findings underscore the value of user- and context-centered design in uncovering the pragmatic, systems-level needs of end users in a complex primary care service-delivery environment.

## Introduction

Globally, the prevalence of mental illness is rising, with approximately 1 billion people living with some form of mental illness in 2019, most commonly depression or anxiety [1]. The COVID-19 pandemic had a significant and well-documented impact on global mental health, with initial estimates from the World Health Organization showing a 26% increase in anxiety disorders and a 28% increase in major depressive disorders in 2020 alone [2], placing additional strain on already overstretched mental health services. Despite growing demand, access to adequate care remains limited due to chronic underinvestment in mental health services and ongoing workforce shortages [1]. For decades, international health bodies have highlighted the growing disparity between the demand for mental health support and the available resources, a crisis that has only worsened over time [3]. This treatment gap, reaching as high as 90% in some nations, underscores a persistent global failure to prioritize mental health, leaving public health systems overwhelmed and unable to provide necessary care for a substantial portion of the population [1].

This international trend is evident in New Zealand, where there has been a sharp increase in psychological distress across the population in recent years [4,5]. Demand for mental health services continues to grow, placing significant strain on an already underfunded and resource-limited public health system [6]. This issue is particularly acute among young people, women, Māori, and Pacific peoples, who are disproportionately affected and experience higher rates of psychological distress. Furthermore, socioeconomic status is a significant factor, with individuals in more deprived areas facing a greater likelihood of mental health challenges, compounded by unequal access to care [5].

To address this significant gap in service access, the New Zealand government introduced the Integrated Primary Mental Health and Addictions (IPMHA) model in 2017. This integrated care initiative, based on the primary care behavioral health approach [7], aims to provide accessible, publicly funded care for those with mild-to-moderate mental health and behavioral issues within primary care settings. Following a successful pilot, the IPMHA model was implemented nationwide and, as of mid-2024, was available to over 70% of the enrolled primary care population [8]. The service is delivered by clinicians known as health improvement practitioners (HIPs) who offer brief, 30-minute sessions using focused acceptance and commitment therapy (fACT) [9]. While designed to be delivered within a single session, the model allows for follow-up appointments as needed, with primary care physicians maintaining overall responsibility for patient care [9]. A step-by-step breakdown of a typical fACT session is available in Multimedia Appendix 1. Evaluations have shown that the IPMHA model has improved access to mental health support, particularly for underserved groups, by reducing wait times and minimizing financial barriers [9].

Despite these successes, the IPMHA model faces significant implementation challenges. Workforce shortages and inconsistent application across different regions can lead to unpredictable care quality and availability, and practitioners often struggle to maintain fidelity to the brief, single-session intervention model [9]. While the model has improved mental health service access in New Zealand, the operational efficiency and potential for scalability of the IPMHA model are undermined by these service-delivery challenges.

Digital mental health tools (DMHTs), from self-help apps to technologies that support practitioner-led care, are increasingly seen as a solution to the global mental health treatment gap [10,11]. Although commonly associated with self-guided therapy, the primary promise of these tools is their potential for integration into existing health care services [12-14]. Specifically, embedding a custom-designed digital tool within a service that faces delivery challenges, such as the IPMHA model, could enhance the service’s effectiveness and scalability [9,10,15,16].

While digital tools can be effective alone, their impact is greatest when combined with practitioner support [10,11,14,17]. This suggests that digital mental health solutions should be viewed not only as a way to replace in-person services but also to augment them, thereby improving the efficiency and reach of existing, evidence-based, human-delivered care.

Despite the potential of digital mental health tools, real-world implementation is hindered by low adoption among practitioners [16,18-20]. Several recent systematic reviews show that clinicians are less likely to adopt tools that are difficult to use, increase workload, disrupt workflows, or are poorly integrated with existing organizational systems [18,20-23]. Rather than being conceptualized as stand-alone products, digital tools for integration into existing services should be designed as integral components of the service they are intended to support. Adopting a context-specific, user-centered software development methodology is necessary to ensure that these tools provide practical value while also integrating seamlessly into clinical workflows and organizational systems [10,13,15,16,24].

This study reports on the final phase of a larger research project that used the 5-stage Design Thinking framework [25,26] to develop a DMHT to enhance the IPMHA model. The Design Thinking methodology was selected for this project due to its iterative user-centered approach and proven efficacy in creating effective digital solutions for complex health care service-delivery problems [27,28].

The aim of this study was to evaluate the usability and acceptability of the software minimum viable product (MVP) developed through this process, with a focus on integration into existing primary care systems and workflows. Specifically, the study sought to evaluate the usefulness and usability of the software’s features and to identify whether these features support practitioner adoption and effective integration into the IPMHA service-delivery context.

## Methods

### Research Design and Framework

**Figure 1.**
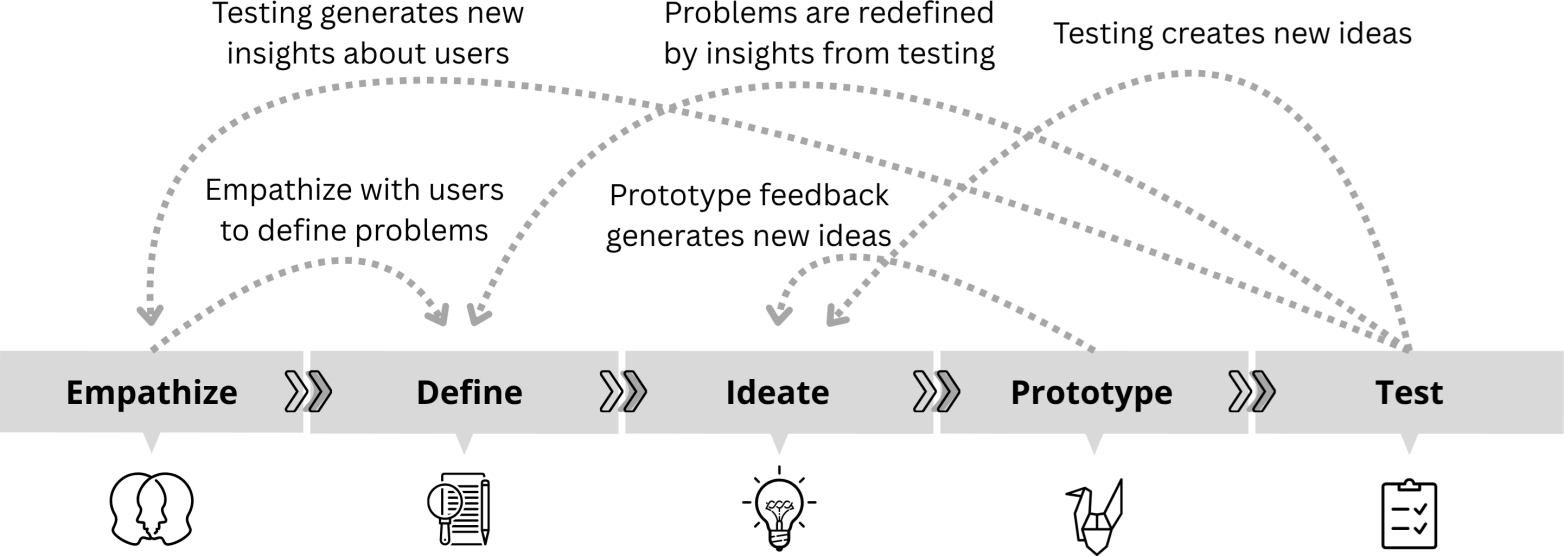
The nonlinear nature of the 5-stage Design Thinking framework. Adapted from Dam [29].

This study reports on the Test stage of the 5-stage Design Thinking framework (Figure 1). The preceding 4 stages, described below, informed the development of the software MVP evaluated in this study.

1. Empathize: The project began with an exploration of HIPs’ experiences working within the IPMHA model through interviews and a workforce survey. This process uncovered a variety of service-delivery challenges, many of which could be addressed by a purpose-built DMHT [30].
2. Define: Service delivery challenges identified during the Empathize stage were translated into user stories and problem statements, ensuring that the project remained focused on validated, contextually relevant, real-world problems.
3. Ideate: The research team generated a broad range of potential software solutions and features based on the problem statements synthesized during the Define stage.
4. Prototype: Software interface prototypes were developed from promising Ideate stage design concepts. Iterative feedback from HIPs and other IPMHA stakeholders shaped the evolution of low-fidelity pen-and-paper sketches into functional wireframe prototypes. The functional wireframes were created using the Figma software package [31]. The culmination of the Prototype stage was the development of a functional software MVP, built using the Flutter programming language [32] for use on an Android tablet.

The purpose of this software MVP was to address the various administrative and workflow challenges reported by HIPs and enhance the delivery of the brief fACT intervention package. To provide a general overview of the software MVP’s functionality, Multimedia Appendices 2-4 present screenshots and interface flows for several key features. While this is not an exhaustive list of the software’s functions, these examples highlight several core capabilities. Multimedia Appendix 2 depicts the user flow for starting a session, which includes selecting a client from the integrated electronic health record, reviewing prior session information, starting a new session, taking notes, and using a model fidelity support tool. Multimedia Appendix 3 details the creation of a wellness plan, and Multimedia Appendix 4 demonstrates the in-session experiential exercise feature.

### Participants and Recruitment

Participants were HIPs working within the IPMHA model in New Zealand primary care clinics. All participants were employed by the same primary health organization (PHO) to ensure consistency in organizational systems and processes. HIPs were recruited through an electronic flyer distributed internally within the participating PHO. Detailed information outlining the study’s purpose and procedures was provided to all prospective participants.

A total of 5 HIPs completed the usability interviews. Small-group user testing is considered appropriate for early-stage software evaluation with relatively homogeneous user groups, where the primary goal is to collect qualitative insights rather than achieve statistical validation. Feedback from 5 participants can uncover approximately 85% of major usability issues, as common problems typically become evident within the first few sessions, with additional participants offering diminishing returns [33-35]. Given the homogeneity of the target user group and the focused, deductive approach to data collection and analysis, small-group testing with 5 participants was deemed appropriate for this study.

Participants were eligible for inclusion in the study if they were aged 18 years or older, were able to provide informed consent, and were currently employed as an HIP within the participating PHO.

### Data Collection

Data were collected through semistructured, one-on-one interviews designed to elicit in-depth feedback on the usability and perceived usefulness of the software MVP. All interviews were conducted in person by the primary investigator. Participants were informed that the interviews would take between 1 and 2 hours.

During the interview sessions, participants’ interactions with the software MVP, running on a tablet device, were captured using the device’s integrated screen recording functionality. This was synchronized with an audio recording of the interview to contextualize participants’ comments about usability and usefulness in relation to their actions within the software. The average interview duration was 76 (SD 23.49) minutes, with sessions ranging from 49 to 117 minutes, meeting the recommended duration for a broad usability analysis [35].

### Interview Procedure

The interview process was structured into 4 phases.

1. Preamble and software orientation: The interviewer introduced the participant to the overall software interface, guiding them through its key features. This phase aimed to familiarize participants with the software and to begin identifying any immediate usability issues. Participants were encouraged to verbalize their thoughts and observations while interacting with the software, following the think-aloud [36] data collection method.
2. Mock fACT session: Participants engaged in a role-play exercise in which they assumed the role of a clinician delivering a typical fACT session using the software. The interviewer acted as the client. Both parties were encouraged to remain “in character” as much as possible to simulate the intended clinical use of the software. Throughout the session, participants continued to verbalize their thoughts and critically reflect on the real-world usability and relevance of different software features.
3. Final think-aloud exploration: Following the mock session, participants were invited to freely navigate the software, revisit specific features, and reflect on their experiences. They were encouraged to comment on the ease of use, functionality, and integration of the software within their existing workflow and organizational processes.
4. Blue-sky brainstorming: The interview concluded with an open-ended brainstorming session, during which participants were invited to share any additional thoughts or suggestions about the software. They were encouraged to propose ideas for new features, suggest modifications to existing features, explore how various features could be integrated within their workflows, and reflect on which features would be essential for addressing service delivery challenges and leading to sustainable engagement with the software.

**Figure 2.**
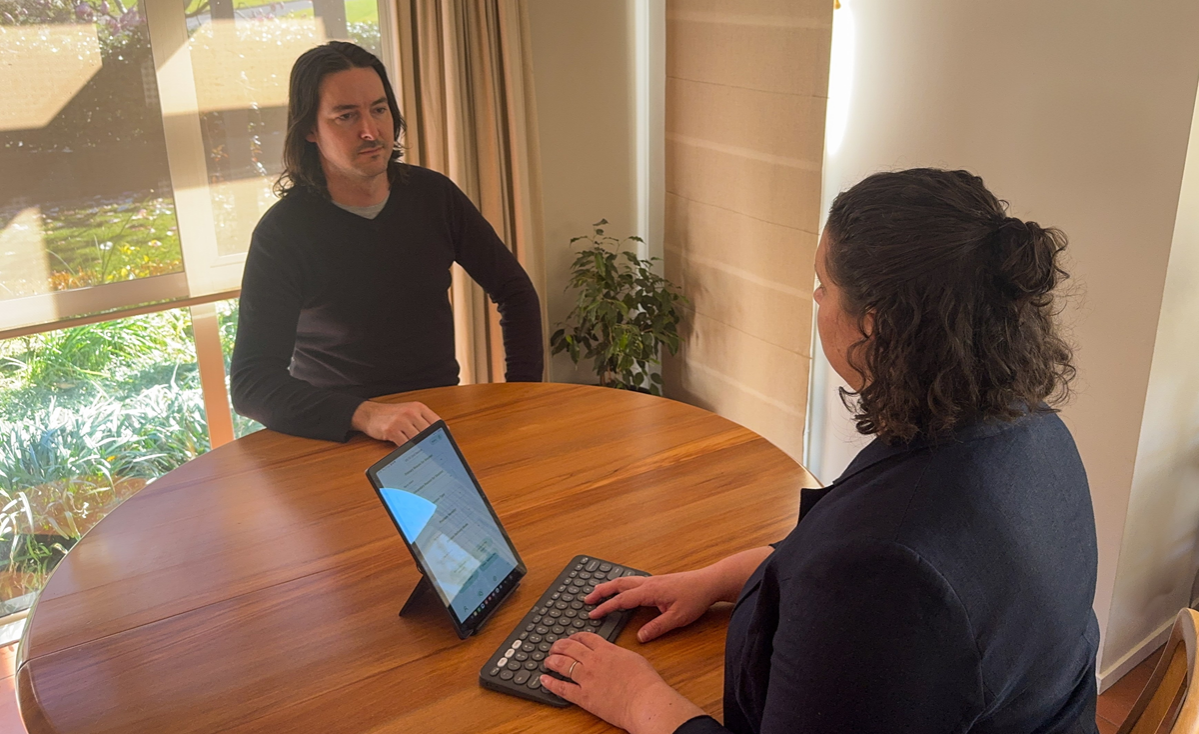
Recreation of the interview setting, showing how the participant engaged with the software minimum viable product (MVP) in a simulated clinical context.

Figure 2 shows the setting for the usability interviews. The participant (substituted here with an actor) is engaging with the software via a tablet and keyboard. The interviewer, who also acted as the “client” during the mock fACT session, sat across from the participant to simulate the clinical context of how the software would likely be used.

During the mock fACT session portion of the interview, participants were instructed to conduct a typical session as they would with a regular client, using the software MVP where appropriate to supplement their standard workflow. This involved the participant using the software to load a client (played by the interviewer) from a simulated clinic database, review client data, take clinical notes, use experiential exercises, create a wellness plan, and commit all notes and session data to a simulated integrated electronic health record upon conclusion.

### Data Analysis

The data analysis methodology chosen for this study was affinity diagramming [37], a qualitative technique commonly used in software development and user experience design [38]. This approach allowed for the systematic exploration of participant feedback concerning the usability and acceptability of the software MVP, particularly in relation to the usefulness, importance, and workflow integration potential of the various software features. The analysis began with a thorough reading of the interview transcripts to gain a high-level overview of the dataset. Each transcript was reviewed alongside the synchronized screen recordings showing participant engagement with the software. Viewing the screen capture alongside participant feedback facilitated a deep understanding of the context surrounding participants’ comments, linking their verbal feedback directly to their interactions with the software.

The data collection process was structured to elicit feedback on specific features of the software, and the software feature set was used as the initial deductive framework for organizing the data. This iterative process involved sorting the interview data into different feature categories and coding relevant interview excerpts (codes) within each category. This included the creation of new categories for any emergent feature-related themes that were identified through the initial reading of the transcripts.

**Figure 3.**
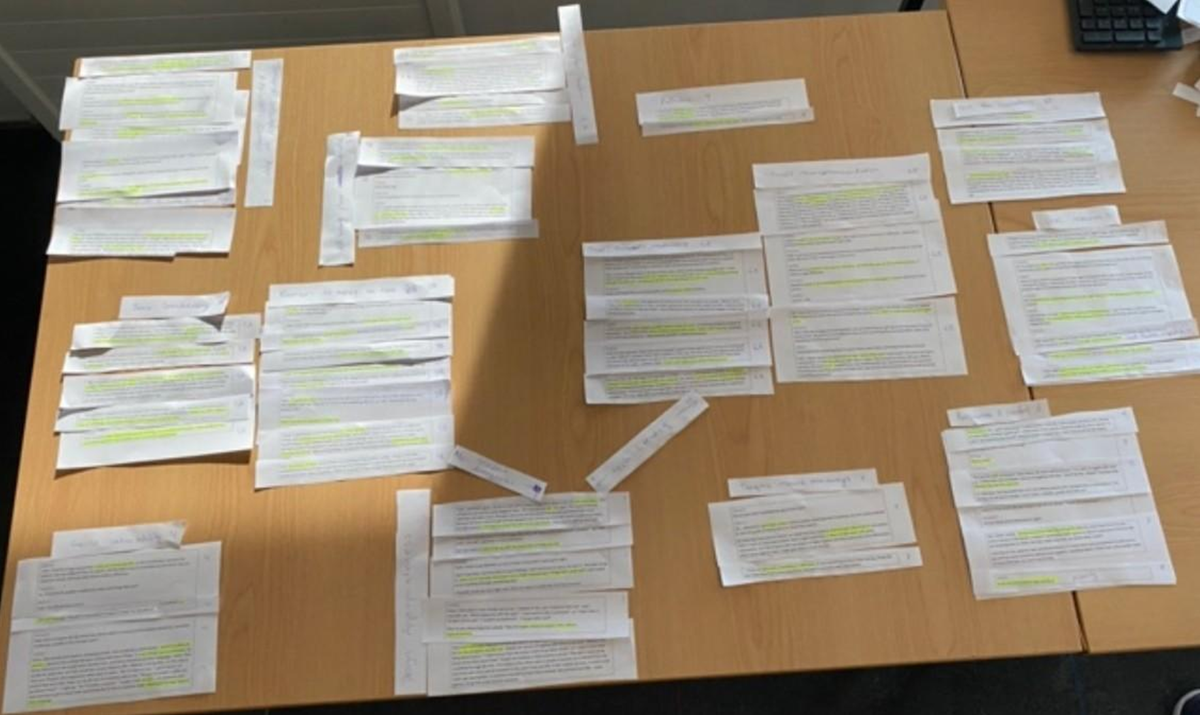
Collaborative affinity diagramming using interview transcript excerpts.

Once the data were categorized and coded, each feature category within the framework was independently analyzed using affinity diagramming to visually organize and group related codes (Figure 3). Related interview excerpts, now represented by these codes, were clustered together into “affinities” These clusters represented overarching themes related to that specific feature. After clustering the codes, each cluster was examined to identify the underlying theme or key message, providing a higher-level summary of user feedback for each feature.

Following the initial feature-driven analysis, an additional analysis of the entire dataset was conducted using the same affinity diagramming methodology. The objective was to identify which features or overall functionalities of the software HIPs identified as being the most important for facilitating effective implementation into workflows and supporting sustained long-term engagement with the software.

The construction of the feature-driven framework, refinement of that framework, transcript coding, and subsequent theme identification were conducted collaboratively by the principal researcher (VA) and a trained research assistant. The Nvivo-14 software package assisted with data organization and tracking, whereas printed copies of the transcripts were used for performing the collaborative affinity diagramming activities.

Collectively, these analyses sought to answer key questions regarding the usefulness of specific features and functionalities, the perceived importance of those features and functionalities, and the potential for integration into the HIP workflows and broader organizational systems. The findings are detailed in the *Results* section, providing a comprehensive understanding of the software MVP’s usability and acceptability from the perspective of its intended users.

### Ethical Considerations

This study was approved by the Auckland Health Research Ethics Committee (reference AH21908). Informed consent was obtained from all participants. Participants received a voucher valued at $50 NZD (US $29.50) for each hour of interview time. During the transcription process, any information relating to participant identity was removed, with a unique participant identifier code assigned to each interview script to ensure confidentiality.

## Results

### Critical Features and Functionalities to Support Practitioner Adoption and Workflow Integration

A primary goal of this research was to identify which software features HIPs deemed most important for successfully integrating the tool into their workflows. This subject was a central focus during the usability interviews. The functionalities that HIPs specified as necessary to support the adoption and effective implementation of the software MVP are detailed in Textbox 1 and elaborated upon in the description that follows.

Participants indicated that their willingness to adopt the software is contingent on its ability to solve existing service-delivery problems and demonstrably reduce their workload. If the tool fails to do this, it will likely be treated as an optional accessory rather than an integral part of their workflow.

The most critical area identified for workflow improvement was the reduction in administrative burden. Key areas included reducing double-handling of data, automating psychometric collection and scoring, and streamlining the process for sharing resources with clients. HIPs reported that these administrative frustrations were compounded by broader IT issues. They described frustrations in dealing with workflow friction caused by software incompatibilities, the need to switch between different devices, and managing multiple logins for various systems. A key benefit of the new software, they emphasized, is its ability to eliminate these technical hurdles.

Textbox 1. **Features and functionalities necessary to support implementation of the software.**

1. Administrative optimization and time-saving
2. Seamless integration with existing practice management software
3. Solving IT and workflow frustrations
4. Must solve problems and reduce workload, not add tasks to an already busy workflow

To solve these interconnected issues, participants consistently stated that seamless integration with existing clinic patient management software was essential. They expressed a strong need for the software to automatically synchronize data, which would minimize manual data entry and ensure a smooth, unified workflow. This was seen as particularly important for practitioners who work across different clinics and PHOs. Ultimately, HIPs indicated that without a high level of systems integration to solve their core administrative and IT problems, they would be unlikely to use the software.

### Usability and Usefulness of Specific Software Features

Participant feedback on specific features is presented in Table 1, organized by software feature or functionality. Feedback included feature validation, constructive critiques, and suggestions for modification. The summary in Table 1 is elaborated upon in the subsequent descriptions, and a detailed point-by-point breakdown is available in Multimedia Appendix 5.

**Table 1.** Practitioner feedback on specific features and overarching functionalities of the software minimum viable product (MVP).

Software feature	Feature validation	Critique and change requests
Experiential exercises and recordings.	Useful for early career and fatigued HIPs^a^. They are well suited for brief sessions. Easy to integrate into existing workflow.	Additional customizability for different client needs. Adding visual elements may improve engagement with recordings.
Love Work Play Health tool.	Enhances model fidelity. Provides a useful reminder for early career HIPs.	The design should encourage flexible use.
Model fidelity checklist.	Enhances model fidelity. Useful for both novice and experienced HIPs.	Would benefit from key information being more accessible. Would benefit from better integration into workflow.
Session history review.	Improved efficiency compared to clinic electronic health record. Provides easy access to patient-specific information and reminders.	—^b^
Automation of external data reporting.	Reduces double-handling of data. Automating psychometric scoring and reporting. Structuring notes to align with reporting needs.	—
Psychometric recording and analysis.	Expanding psychometric options for clinical use. Enhancing psychometric administration in session. Improving workflow and integration with note-taking.	—
Psychometric review.	Visualizing change over time. Supporting client conversations.	—
Session timer.	Time management and session flow.	—
Values elicitation and recording.	—	Efficiency and collaboration. Language and accessibility issues. Integration with contextual interview and note-taking.
Wellness plan creation.	—	Wellness plan creation should be flexible. Terminology concerns. Usability and interface design. Inclusion of a support person.
Clinical note and session data recording.	Reducing administrative burden.	Integrating note-taking with model fidelity tools and overall session flow. Improving access to notes and supporting personal reflections. Automating data recording and reporting.
Integration of the software with existing clinic IT systems.	Integration with clinic patient management software is essential. Reducing administrative burden and double-handling of data. IT simplification across clinics and PHOs^c^. Supporting model fidelity and reliable data collection.	Securely and efficiently sharing resources with clients. Ensuring data security and clinic buy-in.
Postsession support for clients.	Providing clients with session takeaways. Collecting postsession outcome data.	Balance between ongoing support and managing HIP workload. Personalizing and adding value to session summaries. Overcoming administrative challenges. Format of takeaway resources must be flexible around client needs.

a HIP: health improvement practitioner.

b Not applicable.

c PHO: primary health organization.

#### Experiential Exercises and Recordings

Participants felt that having a library of prerecorded experiential exercises was a valuable asset, particularly for early-career or fatigued HIPs who might need support in delivering them consistently. The shorter exercises, such as mindful breathing, were seen as especially well suited for the brief intervention model. HIPs appreciated that having these resources integrated directly into the software was more efficient than searching for content online. Furthermore, the ability to use editable scripts allowed for customization in pacing and duration to better meet individual client needs, such as during moments of panic. To further improve client engagement, participants suggested adding visual elements or animations to the recordings and valued the ability to send recommended exercises to clients as part of a session summary.

#### Love Work Play Health Tool

The Love Work Play Health tool was seen as a valuable feature for enhancing fidelity to the fACT model. Participants reported that it provides a structured guide to ensure a thorough contextual interview is conducted, prompting HIPs to explore key life domains and client values. This was considered particularly useful as a reminder for newer practitioners. However, HIPs stressed the importance of flexibility, noting that it would be impractical to ask every prompt in a single session. They recommended including guidance that clarifies the tool is a flexible aid rather than a rigid script to be followed verbatim.

#### Model Fidelity Checklist

Participants indicated that a digital Model Fidelity Checklist was a practical tool for ensuring correct fACT delivery, serving as a step-by-step guide for novices and a helpful refresher for more experienced HIPs. Many practitioners already use printed reference materials, so integrating a digital checklist directly into the session workflow, for instance, within a note-taking template, was seen as a significant improvement. To enhance usability, participants suggested making key information more accessible through on-screen prompts or expandable info buttons. They also proposed that embedding the checklist directly into the session notes would encourage its active use and could even help streamline external data reporting.

#### Session History Review

The ability to easily review a client’s session history was highlighted as a significant efficiency gain. Participants described the current process of reviewing past notes in clinic patient management software as slow and cumbersome. The proposed feature, which collates previous session notes, allowed HIPs to quickly prepare for appointments, access important client-specific information, and recall personal details that aid in building rapport. Moreover, HIPs often leave themselves reminders in session notes, and easy access to this history would ensure important follow-up tasks are not overlooked.

#### Automation of External Data Reporting

Automating external data reporting was considered an essential time-saving feature by participants. They expressed significant frustration with the “double-handling” of data, where they must reenter the same information for both clinical notes and separate reporting systems. The software’s ability to capture information once and automatically transmit it was seen as a key function that would significantly reduce administrative workload. Likewise, automating the scoring and reporting of psychometric measures would not only save time but also improve model fidelity. Participants suggested that structuring note-taking templates to align with reporting requirements would further streamline this process.

#### Psychometric Recording and Analysis

Participants appreciated the software’s flexibility in offering a range of psychometric tools beyond the 2 mandated ones, noting that practitioners often use other measures, such as the 10-item Kessler Psychological Distress Scale Kessler-10 or 9-item Patient Health Questionnaire, which are more familiar to referring physicians. The ability to administer these assessments collaboratively, either by reading them aloud or having clients complete them, was valued, and the automatic scoring feature was seen as a major time-saver that allows for the immediate discussion of results in-session. HIPs also stressed the need for a seamless workflow, allowing them to pause an assessment to explore a client’s response and skip questions if necessary.

#### Psychometric Review

The ability to visually track a client’s psychometric scores over time was viewed as a highly valuable feature. Participants explained that seeing these changes at a glance helps them monitor progress and identify areas needing further attention. They also noted that presenting these data visually on a single screen would significantly improve their workflow when reviewing results with clients. This direct comparison of past and present scores facilitates meaningful conversations about progress and helps collaboratively identify new goals.

#### Session Timer

A session timer was seen as a simple but effective tool for managing session flow and adhering to tight schedules. Participants noted that having a visible timer benefits both the practitioner and the client. For the HIP, it ensures all components of the fACT model can be covered without feeling rushed. For the client, being able to view the remaining session time during collaborative tasks could help maintain focus.

#### Values Elicitation and Recording

Participants highlighted the need for a more efficient and accessible way to elicit client values, as traditional methods, such as card sorts, can be time-consuming. They suggested that the term “values” can be confusing for clients and recommended using simpler, more relatable language, such as asking, “What is important to you?” While values often emerge naturally during the contextual interview, HIPs expressed a desire for a designated, collaborative space within the software to seamlessly record them as they are identified.

#### Wellness Plan Creation

Flexibility was the key theme for feedback on the wellness plan feature. Participants emphasized that creating a plan should accommodate diverse client needs, with optional fields for elements, such as start dates or reminders. They also raised concerns about terminology, suggesting that words such as “likeliness” or “confidence” are more suitable than “willingness,” and “support person” is preferable to the informal term “buddy.” From a usability perspective, they recommended a more user-friendly interface for setting dates and frequencies. The ability to include a support person was seen as beneficial for client accountability, but it was stressed that clear information about data privacy is essential. Finally, providing a printable summary is crucial for clients who may not engage with digital resources.

#### Clinical Note and Session Data Recording

The primary value of this feature lies in its potential to significantly reduce administrative burden. Participants emphasized that eliminating the “double-handling” of data by entering notes once and having them automatically sync with the clinic’s patient management software was a critical function. They also desired a workflow where fidelity tools are integrated directly into the note-taking process, using structured templates that mirror the natural progression of a session. Quick access to past notes, a separate space for personal reflections, and the ability to attach nontext materials were also identified as valuable enhancements. To ensure proper data management, participants suggested reminders for obtaining client consent and automatic submission of draft notes after a set period to prevent lost records.

#### Integration of the Software With Existing Clinic IT Systems

Across all interviews, seamless integration with existing clinic IT systems, particularly with patient management software, was identified as an essential, nonnegotiable requirement for adoption. Participants stated that the software must solve current administrative problems, not create new ones. By automating data entry, synchronizing notes and psychometrics, and reducing the “IT friction” of multiple logins and incompatible systems, the software would significantly reduce workload and improve job satisfaction. This integration is key to ensuring consistent data collection and allowing HIPs to securely and efficiently share resources with clients.

#### Postsession Support for Clients

Participants valued the software’s ability to extend support beyond the session. They highlighted the importance of providing clients with tangible takeaways, such as wellness plans and experiential exercises, in a format that suits their needs, whether digitally via email or as a printed hard copy. The ability to personalize these summaries with key insights or links to external resources was seen as a way to enhance client engagement. While collecting postsession outcome data via a client-facing app was considered useful, HIPs cautioned that a balance must be struck to avoid overwhelming clients with excessive notifications. Streamlining the process of sending these resources was seen as a key benefit that could overcome common administrative challenges within clinic settings.

## Discussion

### Principal Results

The key objectives of this study were to evaluate the usability and acceptability of the software MVP and identify which features and functionalities were critical for facilitating effective integration into the IPMHA service-delivery context. The analysis revealed 2 nonnegotiable prerequisites for the software’s adoption into HIP workflows: significant administrative optimization and seamless integration with existing IT systems. Participants’ willingness to use the tool was primarily contingent on its ability to reduce their workload by automating tasks, such as note-taking, data reporting, and psychometric scoring. This need for efficiency was directly linked to the second prerequisite: robust integration with existing patient management software to synchronize data, eliminate duplicate entry, and resolve workflow friction. Without these foundational capabilities, participants indicated the software would likely be viewed as a workload-increasing add-on rather than an integral component of their clinical practice.

Beyond the overarching need for efficiency and integration, several individual features were highlighted for their potential benefits. The inclusion of experiential exercise recordings was seen as a valuable resource, particularly for newer or fatigued HIPs. The Love Work Play Health and model fidelity checklist tools were acknowledged for their potential to support HIPs with in-session training, promote adherence to the fACT model, and reinforce best practices in session delivery. The session history review feature was praised for offering a more efficient way to access information from previous client interactions. Finally, the psychometric recording and analysis tools were appreciated for expanding the available assessment options and streamlining their administration within sessions.

These findings highlight that a successful digital tool designed to support HIPs working within the IPMHA model must not only offer features to assist with clinical practice but also seamlessly integrate with existing systems and significantly reduce the administrative burden for HIPs. Effectively addressing these administrative optimization challenges will likely be the primary determinant of practitioner engagement, sustained use, and the tool’s impact on IPMHA model scalability.

### Implications for Design

The feedback from HIPs regarding the importance of administrative optimization and seamless workflow integration led to a redesign of the software’s backend as well as several interface changes. These changes were informed by specific usability feedback. For instance, the call for better integration of clinical note-taking with other in-session tools prompted the redesign of the note-taking tool to create a more unified workflow that matched the clinical note format typically used in primary care services. Other notable changes included a dedicated resource upload feature to facilitate sending PDFs and worksheets to clients. HIPs also stressed that they needed to retain the ability to easily print resources for less-tech-capable clients, which prompted the integration of one-click print functionality for the experiential exercise scripts, wellness plan summaries, and session summaries. Most of the model fidelity and fACT support tools were retained in the same format, with minor changes in response to feedback—for example, the addition of a “support person summary screen” in the wellness plan to enhance transparency regarding what information would be shared.

It is important to view these design changes not as a final step but as part of the ongoing iterative Design Thinking process. Our team is now applying feedback from the usability study to refine the software for clinical implementation. Most of the requested features involve backend modifications to ensure seamless integration with existing clinical systems, requiring only minor changes to the user interface. These backend updates will be incorporated into our next MVP, which will then be tested in a clinical implementation trial to validate its effectiveness and acceptability in a real-world primary care setting.

### Strengths and Limitations

This study has several key strengths. The methodological approach, combining semistructured interviews with a think-aloud protocol and a mock fACT session, yielded rich, in-depth qualitative data. This captured both high-level reflections on the software’s features and direct, in-the-moment usability feedback. A particular strength of this study was its use of a simulated clinical environment. Having the participant actively use the software MVP in a realistic service-delivery context helped uncover usability issues that are often missed by less hands-on methods, such as simple feature walkthrough interviews or focus groups. Furthermore, this study demonstrates the usefulness of rapid, small-sample usability testing in generating rich and actionable insights, highlighting the efficiency of this low-resource approach for iterative development cycles.

By focusing on a homogeneous user group (HIPs from a single PHO), we controlled for organizational variables. This approach provided a clearer understanding of the software’s usability in a specific service context and generated actionable data to guide the MVP’s iterative development for use with this specific group of prospective users.

However, the study also has notable limitations. The most significant limitation is that the usability analysis was not based on real-world clinical usage of the software. Consequently, the findings reflect practitioners’ perceived usability and anticipated usefulness based on a simulated clinical environment, which may not fully correspond to their experiences during actual, day-to-day clinical practice. The small sample size of 5, while appropriate for uncovering major usability issues, limits the generalizability of these findings. Similarly, the recruitment of participants from a single PHO implies that the results may not be generalizable to HIPs working in different organizational or geographical contexts. Finally, there is a potential for social desirability bias, as participants may have been inclined to provide positive feedback to the interviewer, who was also the primary investigator involved in the development of the software.

### Comparisons With Prior Research

To situate the findings of this study within the broader academic literature, we compared the software features and functionalities prioritized by HIPs against the key barriers to practitioner digital adoption identified in prior research. A comprehensive synthesis of 6 major systematic reviews [10,18,19,21-23] reveals a variety of common practitioner digital adoption barriers. Across different health care contexts, a core set of three challenges consistently emerges: (1) the fear that digital tools will increase practitioner workload and disrupt workflows; (2) inadequate training and support with digital tools; and (3) concerns regarding data privacy and security.

The most significant theme to emerge from our study, the demand for administrative optimization, strongly validates what the literature identifies as the most significant adoption barrier: the fear that digital tools will increase practitioner workload [10,18,23,24]. HIPs stated that their adoption of the software hinges on its ability to “solve problems and reduce workload, not add tasks to an already busy workflow.” Specifically, participants valued features that automate clinical notes and data reporting to eliminate the “double-handling” of data, as well as those that automate psychometric scoring to save time. These findings confirm that for IPMHA practitioners, like many other health care professionals, adoption depends on a digital tool demonstrably reducing, rather than increasing, their existing administrative burden.

A common workload-related adoption barrier identified in the literature is system interoperability [10,16,18,19,22,24]. Our second key finding, the necessity of seamless integration with existing practice management software, directly addresses the well-documented barrier of poor system interoperability. HIPs were unequivocal that without robust integration, the software would likely remain unused because it would create, rather than solve, administrative problems. This desire for a high level of interoperability was also reflected in their wish for the DMHT to solve common “IT and workflow frustrations,” such as managing multiple logins and system incompatibilities. These findings confirm that, similar to other digital tools designed for health care implementation [39,40], a DMHT’s value is contingent not on its clinical features alone, but on its ability to integrate seamlessly into existing workflows and organizational systems.

Beyond these primary themes, participants’ concerns also echoed other established barriers. The frustrations HIPs expressed with workflow issues are symptomatic of an environment lacking integrated support systems, aligning with the consensus that effective adoption requires continuous, context-specific training and readily available technical support. Furthermore, the core barrier of privacy and security was also present in our findings. HIPs stressed the importance of “ensuring data security and clinic buy-in” and designing the system with low-risk data handling as a primary consideration. This concern extended to client-facing features, with HIPs noting the importance of clarifying what information a “support person” could access. This confirms that even when a tool offers clear workflow benefits, adoption will be hindered if it does not first establish a foundation of trust and security for both practitioners and the clients they serve.

Interestingly, whereas prior research focusing on mental health tools has highlighted a fear that technology could depersonalize the therapeutic relationship [10,18], our findings reveal a more nuanced perspective from the IPMHA workforce. Participants identified several ways the software could enhance rather than detract from relational aspects of care. For instance, the Session History Review feature was valued for enabling HIPs to recall personal client details to build rapport in subsequent sessions. Likewise, psychometric tools were seen not as impersonal data collection exercises but as a way to facilitate collaborative conversations with clients about their progress. This suggests that for this workforce, when a tool is designed to support, rather than replace, clinical judgment and interaction, it can be perceived as an asset to the therapeutic alliance.

In conclusion, the findings from this study strongly validate well-established evidence from the broader literature. The primary concerns voiced by HIPs—the fear of increased administrative burden and the absolute necessity of seamless workflow and patient management software integration—directly mirror the complex, sociotechnical barriers to digital tool adoption consistently reported across various other health care contexts. This underscores a crucial takeaway: the path to successful implementation of DMHTs is paved by solving the pragmatic, operational challenges that practitioners face. For a digital tool to be adopted by the IPMHA workforce, its value must be demonstrated through its ability to function as a trusted, efficient, and well-integrated component of the complex clinical environment it seeks to support.

### Conclusions and Implications for Future Research

The central message from this study is that, for a digital tool to be adopted by HIPs, its clinical use is secondary to its ability to solve their most pressing administrative and workflow challenges. While clinical support features were valued, they are not the drivers of adoption. Instead, practitioners require a tool that functions as a practical and efficient component of their daily practice, capable of reducing their administrative load through task automation and seamless integration with existing patient management software. These findings suggest that the successful implementation of any DMHT in this context is contingent on its capacity to address the foundational, operational realities of primary care service delivery.

These findings illustrate the value of collaborative and user-centered research methodologies for DMHT development. By grounding the overarching research project in the lived experience of HIPs through a user- and context-centered design framework, this study was able to uncover the true determinants of digital adoption for this group of practitioners. A traditional, top-down, researcher-led approach to digital tool development, which might prioritize theoretical clinical advancements or novel functionalities, would have been at high risk of missing the pragmatic, foundational needs of the end users. Without this direct engagement with end users, the importance of the DMHT in solving administrative and workflow frustrations could have easily been overlooked, leading to the development of a tool that, while perhaps innovative, would ultimately fail to gain traction in the demanding environment of primary care. This underscores that for digital mental health tools to be effective and engaging, their development must be driven by the actual, expressed needs of the people who will be using them.

The findings of this study provide valuable early-stage validation of the required software features and functionalities that a DMHT must possess to effectively support IPMHA practitioners operating within New Zealand primary care. Now that the proposed feature set has been validated, future research will focus on further refinement of the software MVP into a clinically implementable DMHT. This development will prioritize the core administrative and workflow features that were validated in this study, with a particular focus on robust patient management software integration and the automation of practitioner data management tasks. A clinical implementation trial will be conducted in which IPMHA practitioners use the DMHT in their daily practice. The purpose of this trial will be to investigate the tool’s impact on administrative efficiency, assess its usability and feasibility in a live clinical environment, and determine its efficacy in improving client outcomes and enhancing the overall scalability of the IPMHA model.

## Supplementary material

10.2196/84412 Multimedia Appendix 1 Structure of a focused acceptance and commitment therapy (fACT) session.

10.2196/84412 Multimedia Appendix 2 Screenshots of the software minimum viable product (MVP) interface showing electronic health record integration and client selection, session and psychometric review, session start, focused acceptance and commitment therapy (fACT)–specific clinical conversation prompts, and the clinical notepad.

10.2196/84412 Multimedia Appendix 3 Screenshots of the software minimum viable product (MVP) interface showing wellness plan creation, including setting automated plan reminders for the client.

10.2196/84412 Multimedia Appendix 4 Screenshots of the software minimum viable product (MVP) interface showing in-session experiential exercise feature, including voice actor selection, and the exercise script view.

10.2196/84412 Multimedia Appendix 5 Detailed feature-specific user feedback.

## Abbreviations

DMHT: digital mental health tool
fACT: focused acceptance and commitment therapy
HIP: health improvement practitioner
IPMHA: Integrated Primary Mental Health and Addictions
MVP: minimum viable product
PHO: primary health organization
