# Supplementary material for: Evaluating a Digital Mental Health Tool for Implementation Into New Zealand’s Integrated Primary Mental Health and Addictions Service: Usability Study
Source: JMIR Hum Factors. 2026 Jun 4;13:e84412. doi: 10.2196/84412 (PMC13237485; doi:10.2196/84412)
Supplement: Multimedia Appendix 1 [file humanfactors-v13-e84412-s001.docx]

|  |
| --- |
| **Structure of a typical fACT session** |

| 1: Setting the stage/Introduction | A powerful two-minute introduction to introduce the practitioner and fACT model and set the scene for radical behavioral change. |
| --- | --- |
| 2: Psychometric/Wellness measure | Measure to establish baseline client functioning. |
| 3: Contextual interview | A targeted conversation with the client using the ‘Love, Work, Play, Health’ worksheet to understand their current life context, establish what’s important to them (values), and identify a problem that they would like help with today. |
| 4: Functional analysis of the problem | Using the ‘Time, Triggers, Trajectory’ questions to explore the context of the identified problem: When did it start? Is there anything that makes it better or worse? Does it get better or worse over time? |
| 5: Problem severity rating | The client's current assessment of the severity of the problem, rated on a scale of 1 to 10. |
| 6: Summary and agreement | Reaching a consensus with the client regarding the nature of the identified problem and presenting them with various intervention choices to address it. |
| 7: Intervention/Behavior plan development | Developing a behavioral experiment that the client will carry out post-session. |
| 8: Client confidence rating | The client's self-assessment on a scale of 1 to 10 regarding their confidence in executing the plan. |
| 9: Session helpfulness question | The client's assessment, rated on a scale of 1 to 10, of the session's helpfulness. |
