# Supplementary figures and images for: Evaluating a Digital Mental Health Tool for Implementation Into New Zealand’s Integrated Primary Mental Health and Addictions Service: Usability Study
Source: JMIR Hum Factors. 2026 Jun 4;13:e84412. doi: 10.2196/84412 (PMC13237485; doi:10.2196/84412)

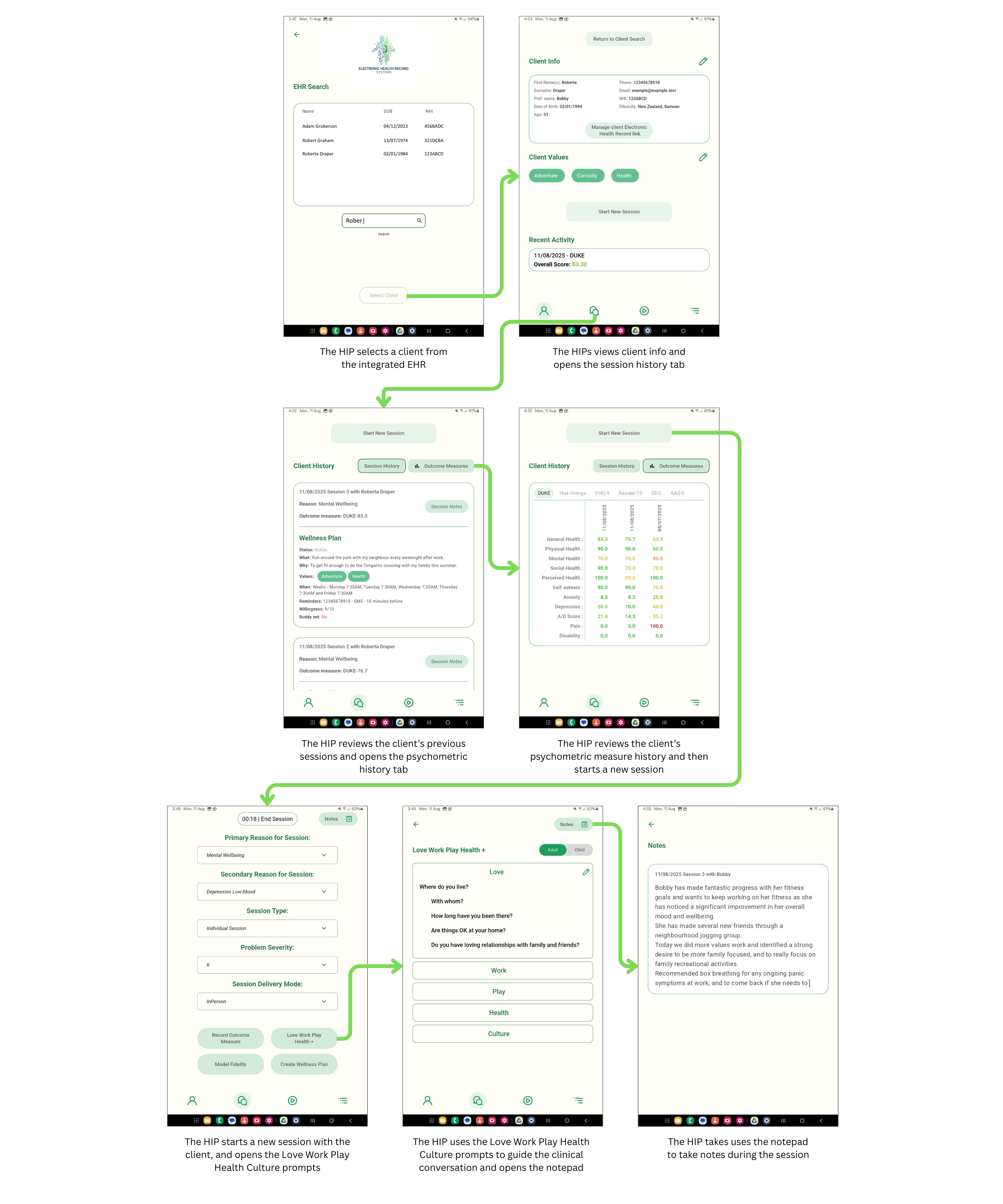

Supplement: Multimedia Appendix 2 [file humanfactors-v13-e84412-s002.png]

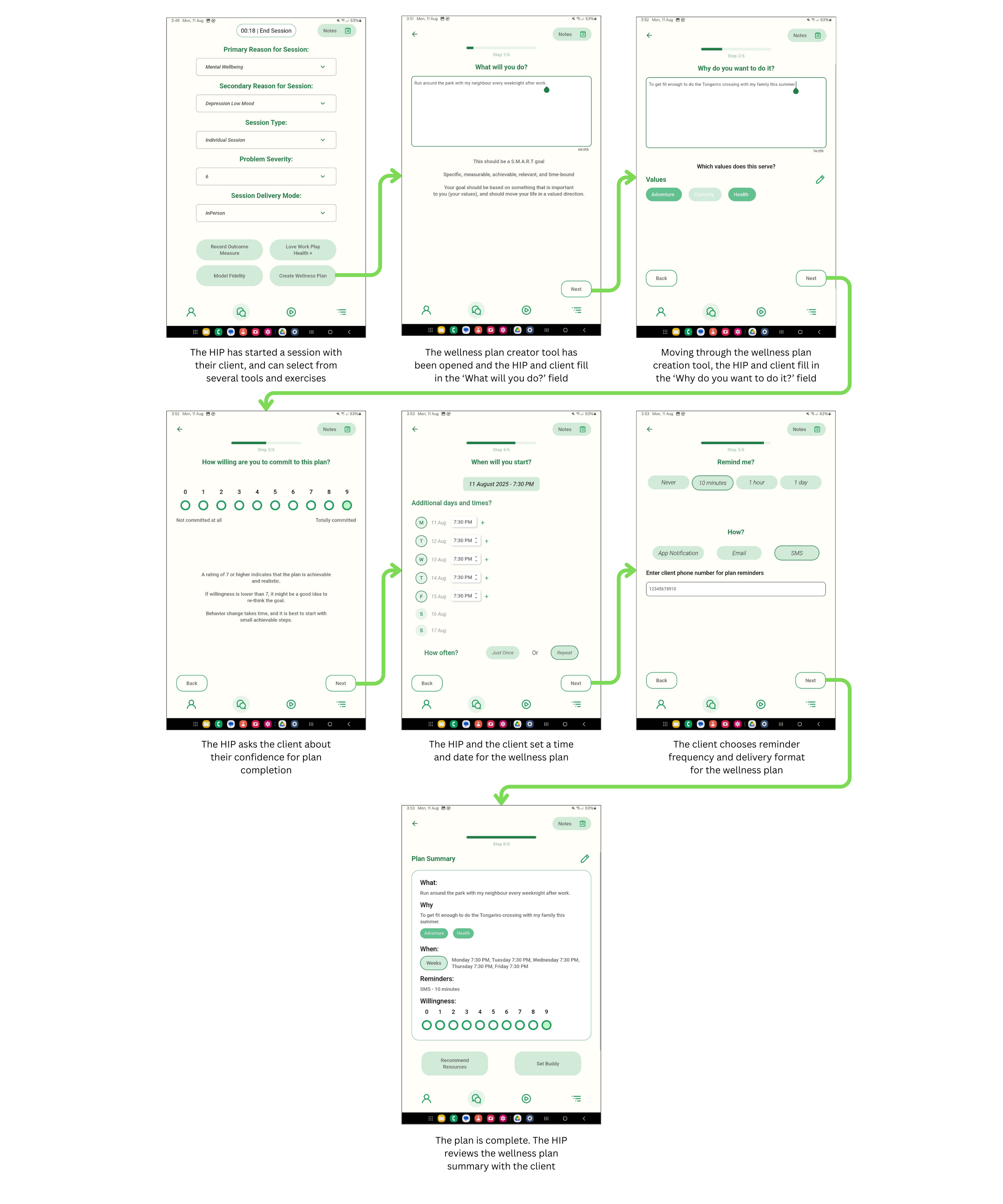

Supplement: Multimedia Appendix 3 [file humanfactors-v13-e84412-s003.png]

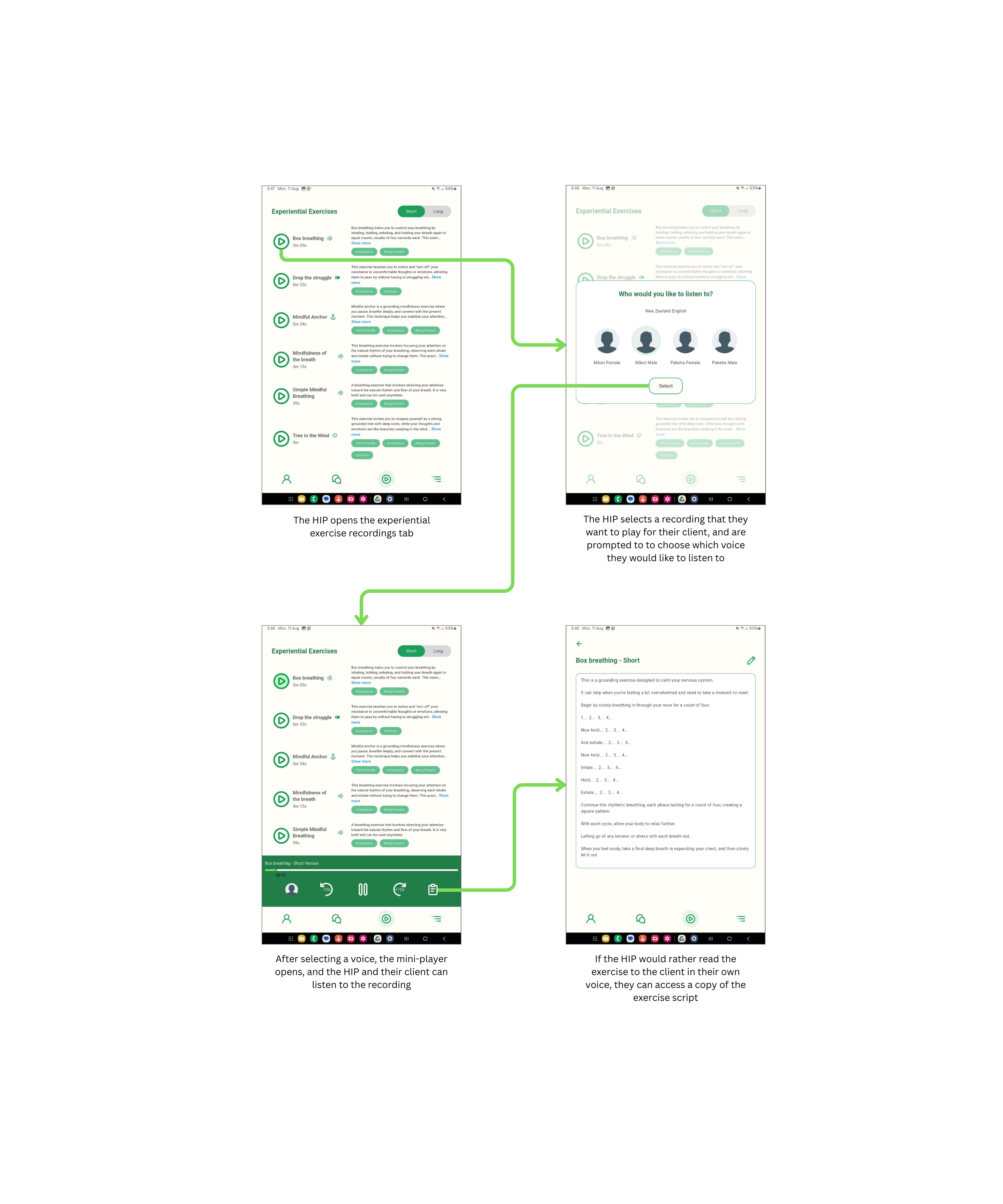

Supplement: Multimedia Appendix 4 [file humanfactors-v13-e84412-s004.png]
