# Supplementary material for: Evaluating a Digital Mental Health Tool for Implementation Into New Zealand’s Integrated Primary Mental Health and Addictions Service: Usability Study
Source: JMIR Hum Factors. 2026 Jun 4;13:e84412. doi: 10.2196/84412 (PMC13237485; doi:10.2196/84412)
Supplement: Multimedia Appendix 5 [file humanfactors-v13-e84412-s005.docx]

# Feature Specific Feedback

***Experiential Exercises and Recordings***

1. Useful for early career and fatigued Health Improvement Practitioners (HIPs):

Participants highlighted several potential benefits of having exercise recordings available. They mentioned that newer HIPs could find these recordings helpful as they might not have memorised any exercises yet. Participants also pointed out that recordings would be a useful resource for any HIP who might be feeling tired, nervous, or uncertain about leading the exercises themselves. Overall, the interviewees felt that access to pre-recorded exercises would support a more consistent delivery of sessions.

1. They are well suited for brief sessions:

The interview participants also noted that the shorter exercise variations are a good fit for brief intervention formats. They indicated a preference for quick and simple exercises, specifically mentioning examples like the mindful anchor and box breathing, over exercises that are more complex.

1. Easy to integrate into existing workflow:

Participants highlighted the efficiency of having readily available resources compared to searching for external content like YouTube videos. They felt that having exercises and scripts directly accessible would make their in-session use much more streamlined and efficient.

1. Additional customizability for different client needs:

Participants also expressed positive views on the flexibility offered by the exercise scripts. They appreciated that the scripts allowed HIPs to adapt the pacing, including modifying pauses and speed to better suit the individual needs of their clients. Several participants specifically mentioned that the ability to customize the duration of breathing exercises would significantly enhance their usability, particularly when working with clients experiencing panic. Moreover, the suggestion of being able to create custom recordings using the HIP's own voice was seen as a valuable feature that could improve personalization and client engagement.

1. Adding visual elements may improve engagement with recordings:

Participants also shared insights regarding visual elements. Some noted that certain clients tend to engage more effectively with visual aids, suggesting that incorporating visuals into some exercises could be beneficial. They also pointed out that the use of icons and structured layouts contributes to improved usability. Regarding video metaphors, there was a suggestion to enhance them further with animations. Lastly, the ability to send recommended exercises to clients as part of a session summary was highlighted as a highly valued feature.

***Love Work Play Health Tool***

1. Enhances model fidelity

Participants reported that the tool would help to ensure HIPs conduct a thorough contextual interview and explore key life domains, reinforcing consistency in model delivery, ensuring that HIPs cover important areas like eliciting values and identifying problem areas.

1. Provides a useful reminder for early career HIPs

Participants indicated that the prompts serve as a valuable refresher, particularly for newer HIPs who may benefit from guidance on appropriate questions to ask. Additionally, they suggested that integrating this tool with note-taking functionalities and other typical in-session activities would further enhance its overall usability.

1. The design should encourage flexible use

Participants acknowledged the usefulness of the prompts but pointed out that asking all of them within a single session would be impractical given time constraints. They suggested that it would be beneficial to include guidance clarifying that HIPs are not expected to ask every single question, emphasizing that the tool is intended to be a flexible aid rather than a rigid script.

***Model Fidelity Checklist***

1. Enhances model fidelity

Participants indicated that the checklist would be helpful in ensuring HIPs deliver fACT correctly and would be particularly useful for less experienced practitioners. Many HIPs already use printed reference materials, suggesting that a digital integration of the checklist would be more practical than having it as a separate document. Furthermore, they felt that embedding this structured guide directly within the session workflow, e.g., within a note-taking template, would reinforce best practices and help highlight any gaps in adherence to the model.

1. Useful for both novice and experienced HIPs

Participants noted that the checklist offers valuable step-by-step guidance for newer HIPs while simultaneously acting as a helpful refresher for more experienced practitioners. They also suggested that having built-in instructions and prompts for various components of the model would make the tool beneficial for HIPs across a range of experience levels.

1. Would benefit from key information being more accessible

Participants mentioned that since some HIPs already utilise prompt lists for note-taking, incorporating similar on-screen prompts or expandable "extra info" buttons within the digital checklist could enhance its accessibility during sessions. Furthermore, participants proposed that the checklist could be expanded to include additional guidance on areas where training may be lacking, such as risk identification, addiction screening, and management strategies.

1. Would benefit from better integration into workflow

Participants suggested that embedding the checklist directly into the session notes would make it more prominent and actively used rather than serving as a separate, passive reference. They also proposed using structured templates within the notes, with headings aligned to the checklist items, as a way to improve usability and adherence to the model. Furthermore, it was noted that this integration could potentially streamline external data reporting, reducing time spent on administrative tasks.

***Session History Review***

1. Improved efficiency compared to clinic electronic health record

Participants highlighted that reviewing past session notes within the clinic patient management software is a slow and cumbersome process, often taking too long to locate relevant information. They emphasized that the ability of the MVP to collate previous session notes represents a significant improvement, enabling HIPs to quickly access relevant data in preparation for an upcoming session.

1. Provides easy access to patient-specific information and reminders

Participants noted that HIPs commonly write reminders to themselves within session notes, such as tasks like sending a client a link to a resource or scheduling a follow-up. They emphasized that being able to easily access past session notes would significantly reduce the risk of these important reminders being overlooked. Additionally, they highlighted the value of being able to look up personal details about the client, such as information about their family, pets, or interests, for building rapport in subsequent sessions. The ability to view all relevant information in a single, easily accessible location was identified as a highly valued aspect of this feature.

***Automation of External Data Reporting***

1. Reduces double handling of data

Participants expressed that they currently spend an excessive amount of time re-entering the same data for various purposes, such as clinical notes and data reporting. They highly valued the software's capability to capture information organically and automatically transmit it, as this would significantly reduce their administrative workload and save time. In fact, they considered this an essential feature for adoption, indicating that HIPs would likely be reluctant to use the software if this functionality were not included.

1. Automating psychometric scoring and reporting

Participants pointed out that many HIPs do not frequently record psychometric outcomes due to a lack of time. They felt that automatic scoring, recording, and reporting of these outcomes would not only save valuable time but also enhance model fidelity by simplifying the process of completing psychometric assessments. As a potential improvement, they suggested that the system could include a function to check if all necessary data has been entered, prompting the HIP to complete any missing information or provide a documented reason for its omission.

1. Structuring notes to align with reporting needs

Participants suggested that aligning the notes section with existing data reporting formats would significantly streamline their workflow, as HIPs would only need to enter information once and it could be replicated across systems as needed. Furthermore, they felt that this structured approach could also provide valuable support for newer HIPs by offering guidance on what information to record and reinforcing best practices in documentation.

***Psychometric Recording and Analysis***

1. Expanding psychometric options for clinical use

Participants highlighted that while the DUKE and Hua Oranga are mandated by Te Whatu Ora, HIPs frequently utilise other tools like the Kessler-10, Edinburgh Postnatal Depression Scale, and PHQ-9, particularly for referrals. They noted that GPs and referring physicians are often more familiar with the Kessler-10 and PHQ-9, making these measures more practical in certain primary care contexts. The participants suggested that while the DUKE and Hua Oranga are required, they may not always be the most suitable tools for all primary care settings, and they liked that the software gave them the option to use a different psychometric if necessary.

1. Enhancing psychometric administration in session

Participants expressed that they appreciate having the flexibility to either read the psychometric assessments aloud to clients or allow them to complete them independently. The automatic scoring capability was identified as a significant time-saving feature, enabling HIPs to discuss the results immediately with clients and explore the implications of the results within the session. Furthermore, HIPs indicated a preference for flexibility in the order of questions, providing an example with the DUKE where some prefer to begin with physical health questions before moving to emotional ones, as this can help ease clients into the assessment. Overall, they emphasized the value of conducting psychometrics collaboratively, transforming it into a discussion rather than simply a data collection exercise.

1. Improving workflow and integration with note-taking

Participants emphasized the need for seamless transitions between psychometric recording and other session tasks. For instance, if a question during a psychometric assessment like the DUKE elicits a strong emotional response, HIPs should be able to easily pause the assessment to explore that with the client before continuing. They also highlighted that psychometric questions should be skippable, recognising that some clients may not be emotionally ready to answer certain questions at a particular time. Participants suggested that the software could include a prompt at the end of the session to remind HIPs to complete the psychometric if it hasn't been done. Finally, they noted the value of having access to a detailed view of individual psychometric questions and responses, as this can reveal important insights into a client's strengths and weaknesses that might be missed by just looking at the overall scores.

***Psychometric Review***

1. Visualizing change over time

Participants expressed that being able to easily see changes in psychometric scores at a glance is highly valuable. They noted that this feature would help them track a client's progress across multiple sessions and identify specific areas that may require further intervention.

1. Supporting client conversations

Participants highlighted that they like to review psychometric results with clients, so having all the relevant data displayed on a single screen with an engaging visual would significantly improve their workflow. They also pointed out that being able to compare past and present scores directly within the session would facilitate discussions about progress and generate prompts for conversations about additional areas that the client would like to work on.

***Session Timer***

1. Time management and session flow

Participants expressed appreciation for the inclusion of timers to help them manage session duration effectively, particularly when working within strict timeframes such as 30-minute appointments. They noted that having prominent timers would be beneficial for both HIPs and clients. For clients, awareness of the time they have left can enhance focus and engagement, while for HIPs, it helps ensure that all necessary steps of the fACT model are addressed without feeling rushed.

***Values Elicitation and Recording***

1. Efficiency and collaboration

Participants expressed a desire for a quicker method to elicit client values, noting that traditional approaches like card sorts can be quite time-consuming. They suggested that a faster, more efficient tool would be beneficial, especially one that allows for collaborative exploration of values with the client. Furthermore, they proposed that grouping suggested values in a structured format could streamline the process of reviewing them with the client, making it a more engaging and interactive experience.

1. Language and accessibility issues

Participants highlighted that the term "values" is often misinterpreted by clients, who may confuse it with moral or family values. To mitigate this, they suggested using simpler language, such as "What is important to you?", to make the concept more relatable and easier to understand. While examples of values can be helpful, they noted that the current suggestions might still not be entirely client-friendly and would benefit from adjustments to improve clarity.

1. Integration with contextual interview and note-taking

Participants pointed out that values are frequently elicited organically during the contextual interview rather than through direct questioning. This natural integration of values into the therapeutic conversation avoids the need for a formal or structured values elicitation question. They suggested that it would be helpful to have a designated space within the software to record these identified values seamlessly, both as part of the notes template and potentially through a structured, collaborative tool that actively engages the client in the process.

***Wellness Plan Creation***

1. Wellness plan creation should be flexible

Participants emphasized that wellness plan creation should offer flexibility, with certain elements being optional, such as the start date and time, to accommodate the diverse needs of patients. They noted that not all wellness plans necessitate specific dates or reminders, and in some instances, an ongoing plan without defined start or end times might be more suitable. Furthermore, they suggested that the process of creating wellness plans should allow for skipping certain sections, recognising that HIPs may not always have the time to complete all fields during a session or that a highly specific plan may not always be necessary. In many cases, a summary email or a basic "what" field might be sufficient.

1. Terminology concerns

Participants expressed that the terms "willingness" and "buddy" could be perceived as potentially confrontational or overly informal by some patients. They suggested using alternatives such as "likeliness" or "confidence," which might be more suitable as they are less likely to be interpreted as internalized or overly personal. Specifically, they recommended replacing "buddy" with a more formal term like "support person" to enhance professionalism and clarity. Additionally, they emphasized the importance of informing the client about what information their support person will have access to, particularly concerning data privacy.

1. Usability and interface design

Participants suggested that the interface for selecting start times and dates could be improved for better user-friendliness. They also recommended adding the ability to set commonly used plan frequencies (e.g., "every day" or "every two days") to enhance efficiency. Furthermore, they highlighted the importance of having a printable summary of the wellness plan available for both the HIP and the client, ensuring that it can be easily referenced outside of the session, especially for clients who are not likely to engage with a digital summary.

1. Inclusion of a support buddy

Participants noted that involving a buddy, such as a parent, friend, counselor, or psychologist, can be beneficial for integrating therapy into the client's daily life and providing continuous support for their wellness plan. They also highlighted that the ability to email wellness plans to both the client and their buddy can foster accountability and maintain client engagement with therapeutic mechanisms of change between sessions. However, participants acknowledged that some clients might be hesitant to add a buddy due to privacy concerns and emphasized the need to provide clear information about what data will be shared with the buddy to ensure their comfort and willingness to use this feature.

***Clinical Note and Session Data Recording***

1. Reducing administrative burden

Participants expressed that manual note-taking and data reporting are too time-consuming, and they really valued the ability to enter clinical notes only once and have them automatically distributed to the necessary systems, such as the clinic patient management software. They emphasized that reducing the double handling of clinical data is a highly valued aspect of the software, and that this would solve a significant service-delivery problem for them. Furthermore, participants highlighted the need for flexibility in note-taking, including the ability to draft, edit, and review notes before they are finalized. While some HIPs prefer to review their notes before committing them to the patient management software, the suggestion of automatic submission after a defined period, like two weeks, was raised as a potential solution to prevent lost records and ensure that clinical data is not lost.

1. Integrating note-taking with model fidelity tools and overall session flow

Participants emphasized that HIPs should not have to navigate away from their notes to access model fidelity guidance; instead, this guidance should be seamlessly integrated directly into the note-taking process. They suggested that utilizing templates and structured input fields could help standardize notes and reinforce adherence to the model. Furthermore, they felt that the flow of note-taking should mirror the natural progression of a session, ensuring that fields such as reason for referral, session type, and problem severity are logically positioned. Finally, they proposed incorporating "Extra info" buttons with relevant contextual prompts, such as Love, Work, Play, and Health, directly within the notes interface rather than on separate screens.

1. Improving access to notes and supporting personal reflections

Participants highlighted the difficulty of reviewing previous session notes in existing software systems and expressed that a system offering quick access to relevant past session data would be highly valuable. They also desired a way to store personal reflections or reminders about clients, such as notes about a pet or a helpful resource, without including this information in the official clinical record. Additionally, participants suggested the ability to attach non-text session materials, such as photos of exercises that are typically done with pen and paper, e.g. the Choice Point, directly to the client’s electronic health record.

1. Automating data recording and reporting

Participants emphasized that clinical notes should be submitted directly into the patient management software in the same way as they would be for a typical primary care appointment, rather than as attachments which are often overlooked. They also suggested that additional reporting data should be integrated into this daily record instead of being stored separately. Finally, participants highlighted the need for a reminder for the HIP to obtain consent from the client for data reporting, proposing a simple checkbox that could appear when recording a new client's session data.

***Integration of the Software with Existing Clinic IT Systems***

While not related to one specific feature, integration with existing IT systems to solve existing administrative problems was mentioned by participants during discussions across multiple features.

1. Integration with clinic patient management software is essential

Participants consistently emphasized that integration with the clinic patient management software to solve admin inefficiencies is crucial for streamlining their workflows. They highlighted that automatically syncing session notes, wellness plans, and psychometrics directly to the patient management software would result in significant time savings. Finally, quick and easy access to previous session notes stored within the patient management software was identified as a valuable aspect for ensuring effective continuity of care.

1. Reducing administrative burden and double handling of data

Participants expressed that manually entering clinical notes and reporting session data is a significant time burden and contributes to decreased job satisfaction. They desire the software to automate processes such as data entry, the scoring of psychometrics, and the tracking of outcome measures to minimize the time spent on tasks that don't directly involve clients. Additionally, they suggested that implementing a standardized note-taking format could enhance consistency and improve the reviewability of notes by referring physicians.

1. IT simplification across clinics and PHOs

Participants pointed out that HIPs often work across various clinics and PHOs, each utilizing different IT systems. They suggested that a well-integrated solution could standardize software requirements, thereby simplifying the workflow for HIPs working across multiple locations.

1. Supporting model fidelity and reliable data collection

Participants highlighted that automatically recording and pushing outcome measures and session data to the clinic’s patient management software would ensure consistency and prevent gaps in data collection. Furthermore, they noted that automating the scoring and reporting of psychometric assessments would improve adherence to the IPMHA model.

1. Securely and efficiently sharing resources with clients

Participants emphasized the need for a quick and secure way to email or print session summaries, wellness plans, and exercises for their clients. They suggested that integration with secure email services within the clinic patient management software would enhance efficiency and eliminate the hassle of managing multiple logins. Furthermore, they highlighted the essential ability to attach and send external PDFs or documents to patients, as HIPs utilise a wide range of resources beyond what might be directly available in the software.

1. Ensuring data security and clinic buy-in

Participants mentioned the importance of data security, noting that clinics and PHOs often have significant concerns in this area. Therefore, they stressed that the system must be designed with low-risk data handling as a primary consideration. Additionally, HIPs desire a seamless flow of data across various systems while ensuring robust security and full compliance with all relevant reporting requirements.

***Post-Session Support for Clients***

1. Providing clients with session takeaways

Participants liked being able to attach experiential exercises to wellness plan summaries, and they emphasized the importance of clients being able to easily access materials to support post-session engagement with therapeutic mechanisms of change. They also highlighted the need for session summaries and wellness plans to be easily shared via email or print, depending on the client's preference. Finally, the idea of incorporating motivational quotes and brief encouraging messages within the app for ongoing support was raised. Participants also noted that some HIPs experience difficulties with clinic email systems, making it essential to streamline the process of sending these post-session resources.

1. Collecting post-session outcome data

Participants mentioned that the use of the client-facing app to collect psychometric outcome measures after sessions is a valuable method for tracking the effectiveness of interventions over time.

1. Balance between ongoing support and managing HIP workload

Participants acknowledged the value of post-session follow-ups but cautioned that care be taken with reminder and notification frequency, as excessive notifications and reminders could be bothersome for some clients.

1. Personalising and adding value to session summaries

Participants expressed appreciation for the ability to incorporate personalized insights, metaphors, or key highlights from the session in follow-up communications with clients. They also highlighted the value of including a free-text area within session summaries to add supplementary resources or recommendations, such as website links or mental health tools, as a way to enhance client engagement and support continuity of care.

1. Overcoming administrative challenges

Participants value the ability to easily send resources, wellness plans, and session summaries directly to clients via email or phone. They highlighted the administrative challenges they often face within clinic settings, such as email account issues and limitations with secure accounts, emphasising that a streamlined and simple process for sending these resources would be very useful.

1. Format of takeaway resources must be flexible around client needs

They also emphasized the importance of being able to efficiently print resources for clients who prefer hard copies. Overall, participants appreciated the potential of the software to empower them to support clients in a way that best caters to their individual needs and preferences.

***Critical Features and Functionalities to Support Practitioner Engagement and Workflow Integration***

1. Administrative optimization and time-saving

Participants emphasized that the most critical factor influencing their adoption of the software is its ability to reduce administrative burdens. Key areas identified for improvement include automating clinical notes and data reporting to eliminate the "double handling" of data, automatically scoring psychometrics to save time, and facilitating easier emailing and printing of resources for clients without the need for multiple logins or separate systems. Ultimately, participants indicated that if the software does not effectively solve these administrative problems, it is unlikely to be used.

1. Seamless integration with existing practice management software

Participants consistently emphasized that seamless patient management software integration is essential. They expressed a strong desire for the software to synchronize with their existing systems to minimise manual data entry and ensure smooth workflows across various clinics and PHOs they might work with. Participants made it clear that if this integration is missing, the software is unlikely to be used regularly as it would add additional tasks in an already busy workflow.

1. Solving IT and workflow frustrations

Participants highlighted that they frequently encounter inefficiencies due to the need for multiple logins, switching between different devices, and dealing with software incompatibilities. They emphasized that a significant benefit of this new software would be its ability to eliminate this IT-related friction and ensure seamless communication and integration between different systems.

1. Must solve problems and reduce workload, not add tasks to an already busy workflow

Participants clearly stated that their adoption of the software hinges on whether it demonstrably reduces their workload, rather than becoming an additional task. They indicated that if the software does not effectively streamline their work processes, it will likely be perceived and used as an optional tool rather than an integral component of their regular practice.
